# Supplementary material for: Multivariate analysis of Iris meda Stapf based on phenological and morphological characteristics
Source: PLoS One. 2025 Dec 1;20(12):e0336783. doi: 10.1371/journal.pone.0336783 (PMC12668513; doi:10.1371/journal.pone.0336783)
Supplement: S1 Table — (DOCX) [file pone.0336783.s001.docx]

**S1 Table.** Cluster formation stages of *Iris meda* accessions.

| Stage | Distance | Cluster1 | Cluster2 | Number of Clusters | New Cluster | Next Stage |
| --- | --- | --- | --- | --- | --- | --- |
| 1 | 4.43 | 23 | 95 | 107 | 23 | 37 |
| 2 | 4.81 | 13 | 85 | 106 | 13 | 38 |
| 3 | 5.04 | 35 | 107 | 105 | 35 | 39 |
| 4 | 5.06 | 9 | 81 | 104 | 9 | 40 |
| 5 | 5.30 | 27 | 99 | 103 | 27 | 43 |
| 6 | 5.46 | 28 | 64 | 102 | 28 | 42 |
| 7 | 5.47 | 36 | 72 | 101 | 36 | 45 |
| 8 | 5.49 | 2 | 38 | 100 | 2 | 55 |
| 9 | 5.53 | 15 | 51 | 99 | 15 | 58 |
| 10 | 5.59 | 21 | 57 | 98 | 21 | 41 |
| 11 | 5.60 | 25 | 97 | 97 | 25 | 44 |
| 12 | 5.64 | 30 | 102 | 96 | 30 | 47 |
| 13 | 5.71 | 1 | 37 | 95 | 1 | 56 |
| 14 | 5.73 | 10 | 46 | 94 | 10 | 46 |
| 15 | 5.74 | 17 | 89 | 93 | 17 | 48 |
| 16 | 5.85 | 32 | 68 | 92 | 32 | 52 |
| 17 | 5.91 | 26 | 98 | 91 | 26 | 49 |
| 18 | 5.92 | 14 | 86 | 90 | 14 | 50 |
| 19 | 5.98 | 33 | 105 | 89 | 33 | 51 |
| 20 | 6.04 | 31 | 103 | 88 | 31 | 60 |
| 21 | 6.10 | 18 | 54 | 87 | 18 | 57 |
| 22 | 6.12 | 20 | 56 | 86 | 20 | 62 |
| 23 | 6.12 | 34 | 106 | 85 | 34 | 61 |
| 24 | 6.14 | 29 | 65 | 84 | 29 | 54 |
| 25 | 6.16 | 19 | 55 | 83 | 19 | 59 |
| 26 | 6.19 | 3 | 39 | 82 | 3 | 66 |
| 27 | 6.21 | 6 | 42 | 81 | 6 | 64 |
| 28 | 6.21 | 24 | 96 | 80 | 24 | 69 |
| 29 | 6.32 | 16 | 52 | 79 | 16 | 53 |
| 30 | 6.34 | 22 | 58 | 78 | 22 | 67 |
| 31 | 6.35 | 8 | 44 | 77 | 8 | 63 |
| 32 | 6.50 | 5 | 41 | 76 | 5 | 68 |
| 33 | 6.63 | 7 | 43 | 75 | 7 | 70 |
| 34 | 6.79 | 4 | 76 | 74 | 4 | 65 |
| 35 | 6.85 | 12 | 48 | 73 | 12 | 72 |
| 36 | 6.93 | 11 | 83 | 72 | 11 | 71 |
| 37 | 7.34 | 23 | 59 | 71 | 23 | 82 |
| 38 | 7.62 | 13 | 49 | 70 | 13 | 78 |
| 39 | 7.73 | 35 | 71 | 69 | 35 | 82 |
| 40 | 7.91 | 9 | 45 | 68 | 9 | 101 |
| 41 | 8.10 | 21 | 93 | 67 | 21 | 74 |
| 42 | 8.11 | 28 | 100 | 66 | 28 | 78 |

**S1 Table.** Continued.

| 43 | 8.14 | 27 | 63 | 65 | 27 | 93 |
| --- | --- | --- | --- | --- | --- | --- |
| 44 | 8.38 | 25 | 61 | 64 | 25 | 90 |
| 45 | 8.58 | 36 | 108 | 63 | 36 | 105 |
| 46 | 8.60 | 10 | 82 | 62 | 10 | 75 |
| 47 | 8.63 | 30 | 66 | 61 | 30 | 81 |
| 48 | 8.86 | 17 | 53 | 60 | 17 | 88 |
| 49 | 8.88 | 26 | 62 | 59 | 26 | 85 |
| 50 | 8.94 | 14 | 50 | 58 | 14 | 74 |
| 51 | 8.97 | 33 | 69 | 57 | 33 | 103 |
| 52 | 9.04 | 32 | 104 | 56 | 32 | 84 |
| 53 | 9.22 | 16 | 88 | 55 | 16 | 88 |
| 54 | 9.29 | 29 | 101 | 54 | 29 | 76 |
| 55 | 9.36 | 2 | 74 | 53 | 2 | 87 |
| 56 | 9.38 | 1 | 73 | 52 | 1 | 92 |
| 57 | 9.39 | 18 | 90 | 51 | 18 | 76 |
| 58 | 9.41 | 15 | 87 | 50 | 15 | 80 |
| 59 | 9.43 | 19 | 91 | 49 | 19 | 79 |
| 60 | 9.45 | 31 | 67 | 48 | 31 | 91 |
| 61 | 9.45 | 34 | 70 | 47 | 34 | 79 |
| 62 | 9.47 | 20 | 92 | 46 | 20 | 84 |
| 63 | 9.52 | 8 | 80 | 45 | 8 | 77 |
| 64 | 9.55 | 6 | 78 | 44 | 6 | 77 |
| 65 | 9.60 | 4 | 75 | 43 | 4 | 73 |
| 66 | 9.67 | 3 | 40 | 42 | 3 | 73 |
| 67 | 9.68 | 22 | 94 | 41 | 22 | 86 |
| 68 | 9.76 | 5 | 77 | 40 | 5 | 83 |
| 69 | 9.79 | 24 | 60 | 39 | 24 | 93 |
| 70 | 9.94 | 7 | 79 | 38 | 7 | 87 |
| 71 | 10.26 | 11 | 47 | 37 | 11 | 80 |
| 72 | 10.53 | 12 | 84 | 36 | 12 | 96 |
| 73 | 11.60 | 3 | 4 | 35 | 3 | 83 |
| 74 | 13.11 | 14 | 21 | 34 | 14 | 75 |
| 75 | 14.64 | 10 | 14 | 33 | 10 | 90 |
| 76 | 14.95 | 18 | 29 | 32 | 18 | 81 |
| 77 | 14.96 | 6 | 8 | 31 | 6 | 85 |
| 78 | 15.37 | 13 | 28 | 30 | 13 | 95 |
| 79 | 15.74 | 19 | 34 | 29 | 19 | 89 |
| 80 | 15.80 | 11 | 15 | 28 | 11 | 91 |
| 81 | 17.14 | 18 | 30 | 27 | 18 | 86 |
| 82 | 17.18 | 23 | 35 | 26 | 23 | 101 |
| 83 | 17.59 | 3 | 5 | 25 | 3 | 98 |
| 84 | 18.04 | 20 | 32 | 24 | 20 | 89 |

**S1 Table.** Continued.

| 85 | 18.62 | 6 | 26 | 23 | 6 | 92 |
| --- | --- | --- | --- | --- | --- | --- |
| 86 | 18.73 | 18 | 22 | 22 | 18 | 99 |
| 87 | 18.76 | 2 | 7 | 21 | 2 | 98 |
| 88 | 19.48 | 16 | 17 | 20 | 16 | 97 |
| 89 | 19.81 | 19 | 20 | 19 | 19 | 94 |
| 90 | 19.86 | 10 | 25 | 18 | 10 | 95 |
| 91 | 19.93 | 11 | 31 | 17 | 11 | 96 |
| 92 | 20.61 | 1 | 6 | 16 | 1 | 94 |
| 93 | 20.74 | 24 | 27 | 15 | 24 | 97 |
| 94 | 21.29 | 1 | 19 | 14 | 1 | 102 |
| 95 | 21.34 | 10 | 13 | 13 | 10 | 99 |
| 96 | 23.58 | 11 | 12 | 12 | 11 | 103 |
| 97 | 23.72 | 16 | 24 | 11 | 16 | 100 |
| 98 | 24.73 | 2 | 3 | 10 | 2 | 104 |
| 99 | 25.25 | 10 | 18 | 9 | 10 | 100 |
| 100 | 25.40 | 10 | 16 | 8 | 10 | 102 |
| 101 | 26.28 | 9 | 23 | 7 | 9 | 105 |
| 102 | 26.55 | 1 | 10 | 6 | 1 | 104 |
| 103 | 27.55 | 11 | 33 | 5 | 11 | 107 |
| 104 | 32.21 | 1 | 2 | 4 | 1 | 106 |
| 105 | 35.12 | 9 | 36 | 3 | 9 | 106 |
| 106 | 38.00 | 1 | 9 | 2 | 1 | 107 |
| 107 | 47.29 | 1 | 11 | 1 | 1 | -- |

Distance type: Euclidean.
