# Supplementary material for: Multivariate analysis of Iris meda Stapf based on phenological and morphological characteristics
Source: PLoS One. 2025 Dec 1;20(12):e0336783. doi: 10.1371/journal.pone.0336783 (PMC12668513; doi:10.1371/journal.pone.0336783)
Supplement: Attachment — Submitted filename: Comments.docx [file pone.0336783.s003.docx]

**Comments to be addressed**

The manuscript “Multivariate analysis of *Iris meda* Stapf based on phenological and morphological Characteristics” is scientifically sound and clearly presented.

Following correction to be incorporated in revised MS.

**Abstract**

- Suggestion: Start with the importance of Iris, then summarize findings in 2–3 clear sentences.

**Introduction**

- Some phrasing is repetitive: “important ornamental plant with wide diversity” → “a diverse ornamental species of global importance.”
- Replace: “Iris has been cultivated since ancient times” with “Iris has been cultivated for centuries.”

**Materials and Methods**

- Ensure **past tense** (e.g., “data were collected,” not “data are collected”).

**Results**

- “Genotypic variance was higher than phenotypic variance, indicating genetic control” → split into two:
  1. “Genotypic variance exceeded phenotypic variance.”
  2. “This indicates strong genetic control.”
- “Iris accession IC-203 recorded the highest chlorophyll content” instead of “The highest chlorophyll content was recorded in IC-203.”

**Discussion**

- “The results are in conformity with earlier findings in Iris and related ornamentals, where wide variability was reported by Singh et al. (2019), Kumar et al. (2021), and Ali et al. (2022).”
  - Suggested: “Similar variability has been reported in Iris and related ornamentals (Singh et al., 2019; Kumar et al., 2021; Ali et al., 2022).”
- Use linking phrases to improve flow: “In contrast,” “Similarly,” “This agrees with,” etc.

**Conclusion**

- “The study demonstrated significant variability… which could be exploited in breeding programs and germplasm conservation.”
- Suggested: “Significant variability in Iris germplasm was identified, offering valuable scope for breeding and conservation programs.”

Reference

Add some **latest** reference in material & methods and discussion section as follows:

- Meena, R., Chaudhary, M.K., Gurjar, P.S. et al. Morphological diversity assessment in date palm (Phoenix dectylifera L.) germplasms grown under hot arid region of India. BMC Plant Biol **25**, 1159 (2025). https://doi.org/10.1186/s12870-025-07194-2
- Yadav, L.P., Gangadhara, K., Singh, A.K. et al. Genetic variability, morphological diversity, and antioxidant potential in gynoecious Coccinia accessions: implications for breeding and biofortification. BMC Plant Biol **25**, 844 (2025). https://doi.org/10.1186/s12870-025-06335-x
- Yadav, L.P., Gangadhara, K., Singh, A.K. et al. Genetic diversity, morphological and quality traits of Momordica dioica. Sci Rep **14**, 30241 (2024). https://doi.org/10.1038/s41598-024-81828-7
- Singh, A.K., Yadav, L.P., K, G. et al. Genetic diversity assessment of bael (Aegle marmelos) varieties using morphometric, yield, and quality traits under semi-arid conditions. BMC Plant Biol **25**, 1143 (2025). https://doi.org/10.1186/s12870-025-06985-x
- Yadav, L.P., Koley, T.K., Tripathi, A. et al. Antioxidant Potentiality and Mineral Content of Summer Season Leafy Greens: Comparison at Mature and Microgreen Stages Using Chemometric. Agric Res **8**, 165–175 (2019). <https://doi.org/10.1007/s40003-018-0378-7>
