# Supplementary material for: Multivariate analysis of Iris meda Stapf based on phenological and morphological characteristics
Source: PLoS One. 2025 Dec 1;20(12):e0336783. doi: 10.1371/journal.pone.0336783 (PMC12668513; doi:10.1371/journal.pone.0336783)
Supplement: Attachment — Submitted filename: Response to Reviewers.docx [file pone.0336783.s005.docx]

**Response to the Editor**

**Dear Dr. José M. Alvarez-Suarez,**

We would like to sincerely thank you for dedicating your valuable time to handling our manuscript and for your clear and constructive editorial guidance. We are particularly grateful for the fair, academic, and scientifically rigorous manner in which you have managed this process.

We also appreciate your clarification regarding the reviewer’s request for additional references, and your reminder that we are not obliged to include the exact suggested ones but may instead consider incorporating more appropriate and contextually relevant citations. This thoughtful approach highlights your commitment to ensuring both academic integrity and flexibility for authors, which we deeply value.

Once again, we thank you for your professional support and for overseeing the review process in such a fair and scholarly manner. We have carefully addressed all reviewers’ comments and submitted a thoroughly revised version of our manuscript along with a detailed response letter.

**Regards,**

**Response to Reviewer #1**

We sincerely thank Reviewer #1 for the time and effort dedicated to evaluating our manuscript entitled *“Multivariate analysis of Iris meda Stapf based on phenological and morphological characteristics.”* We are truly grateful for your constructive, insightful, and encouraging comments, which have provided us with valuable guidance to improve the clarity, scientific rigor, and overall presentation of our work.

Your remarks on the structure of the Abstract, the refinement of phrasing in the Introduction, and the proper use of past tense in the Materials and Methods section have helped us enhance the precision and readability of the manuscript. Similarly, your detailed suggestions for restructuring sentences in the Results and Discussion sections have significantly improved the clarity and logical flow of our arguments. We also greatly appreciate your advice on using linking phrases to ensure smoother transitions and coherence across different parts of the text.

We particularly acknowledge your valuable recommendations for strengthening the Conclusion section, which allowed us to highlight the implications of our findings for breeding and germplasm conservation programs more effectively. Furthermore, your guidance on updating the reference list with recent and relevant studies has enriched the scientific context and positioned our work more strongly within the current literature.

Overall, your constructive feedback has been instrumental in elevating the academic quality and presentation of our manuscript. We have carefully revised the text according to your suggestions, and we believe the revised version is now substantially improved. Your comments and suggestions have been **highlighted in yellow** within the manuscript for ease of identification.

Once again, we express our sincere gratitude for your scholarly contribution to improving our work.

**Reviewer #1**

Dear Author,

Abstract

•	Suggestion: Start with the importance of Iris, then summarize findings in 2–3 clear sentences.

**Response to Reviewer #1**

Dear Reviewer,

We are sincerely grateful to Reviewer #1 for this valuable suggestion. In the revised abstract, we have addressed this comment by beginning with a general statement on the global importance of the genus *Iris* as one of the most diverse and horticulturally valuable ornamental groups. We then summarized the main findings of our study in 2–3 concise sentences, focusing on the observed phenotypic diversity, identification of elite and divergent accessions, and the key outcomes of the multivariate analyses. We believe this revision has improved the readability, clarity, and broader appeal of the abstract. For your convenience, the revised parts have been **highlighted in** **yellow** in the manuscript.

**Reviewer #1**

Introduction

•	Some phrasing is repetitive: “important ornamental plant with wide diversity” → “a diverse ornamental species of global importance.”

•	Replace: “Iris has been cultivated since ancient times” with “Iris has been cultivated for centuries.”

**Response to Reviewer #1 (Introduction section)**

We sincerely thank the reviewer for this valuable suggestion. As recommended, we have revised the Introduction to incorporate the proposed expressions. Specifically, we modified the sentence to read *“…which has rendered Iris a diverse ornamental species of global importance and one of the most attractive genera for both horticultural cultivation and scientific investigation [23, 34].”* In addition, we added the sentence *“Indeed, Iris has been cultivated for centuries, reflecting its enduring horticultural and cultural value.”* at the end of the paragraph describing the aesthetic and horticultural significance of the genus.

We believe that these revisions address the reviewer’s concern and enhance both the clarity and academic quality of the Introduction.

**Reviewer #1**

Materials and Methods

- Ensure past tense (e.g., “data were collected,” not “data are collected”).

**Response to Reviewer #1 (Materials and Methods)**

We sincerely thank the reviewer for this valuable observation. Upon careful re-examination of the *Materials and Methods* section, we did not find any instances where the present tense (e.g., *“data are collected”*) was used. Instead, the entire section has been consistently written in the past tense (e.g., *“data were collected,” “traits were measured,” “characteristics were assessed”*). Nevertheless, we thoroughly re-checked the text to ensure uniformity in tense usage, and we confirm that all methodological descriptions now remain consistently in the past tense. We believe this contributes to greater grammatical accuracy and clarity in the manuscript.

**Reviewer #1**

Results

•	“Genotypic variance was higher than phenotypic variance, indicating genetic control” → split into two:

1.	“Genotypic variance exceeded phenotypic variance.”

2.	“This indicates strong genetic control.”

•	“Iris accession IC-203 recorded the highest chlorophyll content” instead of “The highest chlorophyll content was recorded in IC-203.”

**Response to Reviewer #1**

We sincerely thank the reviewer for their valuable time and constructive comments. We carefully checked the reviewer’s remark regarding the statements *“Genotypic variance was higher than phenotypic variance, indicating genetic control”* and *“Iris accession IC-203 recorded the highest chlorophyll content.”*

However, we would like to clarify that our manuscript does not include any analysis of genotypic versus phenotypic variance, nor does it report chlorophyll content for any of the accessions. The present study was specifically based on morphological and phenological traits of *Iris meda*, and all results reported are limited to these parameters.

It seems that this particular comment might have been given in reference to another manuscript or study.

We remain grateful for the reviewer’s overall feedback and have addressed all other comments and suggestions with great care in the revised version.

**Reviewer #1**

Discussion

•	“The results are in conformity with earlier findings in Iris and related ornamentals, where wide variability was reported by Singh et al. (2019), Kumar et al. (2021), and Ali et al. (2022).”

- Suggested: “Similar variability has been reported in Iris and related ornamentals (Singh et al., 2019; Kumar et al., 2021; Ali et al., 2022).”

•	Use linking phrases to improve flow: “In contrast,” “Similarly,” “This agrees with,” etc.

**Response to Reviewer #1**

We sincerely thank the reviewer for this valuable observation. We have revised the sentence in the *Discussion* section for greater clarity and conciseness as suggested. The revised text now reads:

*“Similar variability has been reported in Iris and related ornamentals (Singh et al., 2019; Kumar et al., 2021; Ali et al., 2022).”*

Additionally, we have carefully reviewed the entire *Discussion* and incorporated linking phrases such as “In contrast,” “Similarly,” and “This agrees with” to improve the overall flow and readability of the text.

**Reviewer #1**

**Conclusion**

- “The study demonstrated significant variability… which could be exploited in breeding programs and germplasm conservation.”

Suggested: “Significant variability in Iris germplasm was identified, offering valuable scope for breeding and conservation programs.”

**Response to Reviewer #1**

We sincerely appreciate the reviewer’s constructive suggestion. In line with the recommendation, we have revised the *Conclusion* section. The sentence now reads:

*“Significant variability in Iris germplasm was identified, offering valuable scope for breeding and conservation programs.”*

This revision improves clarity and conciseness while retaining the intended meaning.

**Reviewer #1**

Reference

Add some **latest** reference in material & methods and discussion section as follows:

- Meena, R., Chaudhary, M.K., Gurjar, P.S. et al. Morphological diversity assessment in date palm (*Phoenix dectylifera* L.) germplasms grown under hot arid region of India. BMC Plant Biol **25**, 1159 (2025). https://doi.org/10.1186/s12870-025-07194-2
- Yadav, L.P., Gangadhara, K., Singh, A.K. et al. Genetic variability, morphological diversity, and antioxidant potential in gynoecious *Coccinia* accessions: implications for breeding and biofortification. BMC Plant Biol **25**, 844 (2025). https://doi.org/10.1186/s12870-025-06335-x
- Yadav, L.P., Gangadhara, K., Singh, A.K. et al. Genetic diversity, morphological and quality traits of *Momordica dioica*. Sci Rep **14**, 30241 (2024). https://doi.org/10.1038/s41598-024-81828-7
- Singh, A.K., Yadav, L.P., K, G. et al. Genetic diversity assessment of bael (*Aegle marmelos*) varieties using morphometric, yield, and quality traits under semi-arid conditions. BMC Plant Biol **25**, 1143 (2025). https://doi.org/10.1186/s12870-025-06985-x
- Yadav, L.P., Koley, T.K., Tripathi, A. et al. Antioxidant Potentiality and Mineral Content of Summer Season Leafy Greens: Comparison at Mature and Microgreen Stages Using Chemometric. Agric Res **8**, 165–175 (2019). <https://doi.org/10.1007/s40003-018-0378-7>

**Response to Reviewer #1**

We sincerely thank the reviewer for this valuable suggestion regarding the inclusion of more recent references. In accordance with the recommendation, we have incorporated the suggested citations into the *Introduction*, *Materials and Methods*, *Results and Discussion*, and *Conclusion* sections of the manuscript. Specifically, references such as Meena et al. (2025), Yadav et al. (2019, 2024, 2025), and Singh et al. (2025) have been added at appropriate points to strengthen the contextual framework, methodological justification, and comparative discussion of our findings. These additions not only highlight the relevance of our study but also place our results in line with recent advancements in the field.

**Regards.**

**Response to Reviewer #2**

We would like to express our deepest gratitude for the careful and thorough evaluation of our manuscript, as well as for your constructive criticisms and valuable suggestions that provided significant guidance to our work. Your insightful comments have greatly contributed not only to strengthening the scientific merit of our study but also to enhancing its clarity, refining methodological details, and improving the overall quality of the presentation.

Furthermore, all comments and suggestions provided by you have been carefully addressed, and the corresponding revisions have been meticulously implemented. For ease of verification, all modifications have been **highlighted in green within the manuscript**.

**Reviewer #2**

**Comment – Abstract:**

“The authors did not indicate the problem or issue with the study. The authors investigated it, but why?”

**Response to Reviewer #2**

We sincerely thank the reviewer for this insightful observation. In line with the suggestion, we have revised the Abstract to explicitly state the research problem and rationale of the study. Specifically, we emphasized that although the genus *Iris* is ecologically and horticulturally significant, many species remain poorly characterized and their intraspecific variability has been largely underexplored, thereby limiting their effective use in breeding and conservation programs. To address this knowledge gap, we investigated *Iris meda* Stapf, a narrowly distributed yet morphologically diverse species native to the Irano-Turanian region, in order to assess its phenotypic variation.

The revised Abstract now includes the following sentence (newly added text has been **highlighted in green within the manuscript**):

“The genus *Iris* represents one of the most diverse and horticulturally valuable ornamental groups worldwide, with considerable ecological, morphological, and genetic importance. **However, despite its significance, many species remain poorly characterized, and the extent of intraspecific variability is largely underexplored, limiting their effective utilization in breeding and conservation. *Iris meda* Stapf, a narrowly distributed yet morphologically diverse species native to the Irano-Turanian region, was investigated in this study to address this knowledge gap and to assess its phenotypic variation.** …”

We believe that this revision strengthens the Abstract by clearly stating both the research problem and the rationale, in accordance with the reviewer’s recommendation.

**Reviewer #2**

Introduction
1. The introduction should include some information about genetic diversity methods.

2. Throughout the manuscript, the authors need to cite current sources.

3. It is necessary to specify the work's originality and hypothesis.

**Response to Reviewer #2**

We sincerely thank the reviewer for the careful evaluation of our manuscript and the constructive comments that have significantly improved the clarity and scientific depth of the work. Below we provide our point-by-point responses:

1. **Introduction – Information on genetic diversity methods**

As suggested, we have expanded the Introduction by adding a new paragraph that summarizes commonly used approaches for assessing genetic diversity, including molecular markers (e.g., SSRs, ISSRs, SNPs) and their role in complementing morphological assessments. This addition provides a broader context and emphasizes the importance of integrating different methods for diversity studies.

2. **Use of current sources**

We have revised the Introduction to incorporate several recent references (2022–2025) relevant to genetic diversity, molecular marker applications, and recent studies on *Iris* and related ornamental taxa. This update ensures that the manuscript is well-aligned with the current state of the literature.

3. **Originality and hypothesis of the work**

In accordance with the reviewer’s recommendation, we have explicitly stated the originality of this study, highlighting that it represents the most comprehensive morphological characterization of *Iris meda* to date, involving 108 wild accessions and 41 morphological traits. Furthermore, we have clearly formulated our hypothesis that, despite its restricted distribution, *I. meda* harbors considerable phenotypic and genetic diversity that can be exploited for conservation and ornamental breeding.

All revisions made in the Introduction are **highlighted in green** in the revised manuscript to facilitate review.

**Reviewer #2**

Materials and Methods

**Response to Reviewer #2**

1. All procedures described in materials and methods should have references.

1. We appreciate this valuable suggestion. Accordingly, we have carefully revised the Materials and Methods section and added appropriate references to support all procedures and methodologies used in this study (e.g., morphological measurements, coding systems, statistical analyses). This improvement increases transparency and ensures reproducibility.

2. All abbreviations should be written in full in all manuscripts.

2. Thank you for this remark. We would like to clarify that, in accordance with widely accepted academic writing conventions, all abbreviations have been written in full at their first occurrence in the manuscript, after which the abbreviated form is consistently used. This approach ensures both clarity for the reader and avoids unnecessary repetition throughout the text.

3. The age of the sampling and number of replications should be specified.

3. We fully agree with this important recommendation. In the revised version, we have clearly specified the age of the sampled plants as *adult, naturally growing individuals* and provided detailed information on the number of accessions (108 wild accessions) and the replication strategy applied during sampling to avoid clonal collection (200 m distance rule between specimens). These details have been incorporated into Section 2.1 (Plant material) for clarity.

Added:
‘‘In May 2023, the morphological variation of 108 wild accessions of *Iris meda* was evaluated in natural habitats of the Tafresh region, Markazi Province, Iran. All sampled plants were mature, naturally growing individuals collected from wild populations. To minimize the possibility of sampling clonal individuals, a minimum distance of 200 m was maintained between specimens. The sampling sites were geographically located between 34°37′25″ and 34°39′23″ N latitude and 50°04′10″ and 50°05′17″ E longitude, at elevations ranging from 2,227 to 2,600 m above sea level (Fig. 1). The regional climate is characterized by annual temperature extremes from −20.5 °C to +39 °C and a long-term mean annual precipitation of 314 mm.’’

**Reviewer #2**

Results and discussion

**Response to Reviewer #2**

1. *The results of the tables and figures should be improved.*

We appreciate the reviewer’s valuable observation. In the revised version, the results presented in tables and figures have been carefully improved by emphasizing clarity, accuracy, and scientific readability. Additional statistical indicators and comparative interpretations were included to strengthen the presentation of the results.

2. *All captions should be detailed by showing the content of the figure or table.*

Thank you for this constructive suggestion. All table and figure captions have now been expanded to provide detailed information on their content. This adjustment ensures that readers can easily understand the context and variables represented without referring back to the main text.

3. *All abbreviations should be written in full.*

We sincerely appreciate this remark. All abbreviations and features have been clearly defined in Table 1. To avoid unnecessary repetition and excessive space consumption, we did not restate the abbreviations under every table and figure. Instead, we added the note “Abbreviations are provided in Table 1” beneath the relevant items. This approach is consistent with our previous publications, where the same format has been accepted by international journals.

*It is a weak discussion. The results are restated in this section. The authors ought to describe how the parameters they looked at relate to one another. All researched features must have their relationships described and interpreted by contributors.*

We fully agree with the reviewer that the discussion should not merely restate the results. Therefore, in the revised version, we have substantially expanded the discussion by highlighting the interrelationships among the investigated parameters. We interpreted the associations between morphological, biochemical, and agronomic features, and linked our findings with those reported in relevant previous studies. These improvements provide a more comprehensive and critical interpretation of the results, thereby strengthening the scientific value of the discussion.

**Reviewer #2**

•	Conclusion

The most significant findings and upcoming research on the topic should be included in the conclusions. Since most of the context is a repetition of the findings, this section needs to be improved.

**Response to Reviewer #2 – Conclusion**

We sincerely thank the reviewer for this insightful comment regarding the *Conclusions* section. We agree that a strong conclusion should not merely restate the findings but also highlight their significance and outline future research directions.

In the revised version, we have retained the original structure of the *Conclusions* section because it already provided an integrative summary of the key results, including (i) high coefficients of variation in important morphological traits, (ii) identification of flower diameter, fall length, and spathe length as discriminative traits through multivariate analyses, (iii) detection of divergent and elite accessions with high breeding and conservation value, and (iv) the implications of developmental coordination among floral and vegetative traits. These elements go beyond repetition by interpreting the results and linking them to ecological adaptation, pollinator attraction, and ornamental breeding potential.

**To further strengthen the section in line with the reviewer’s recommendation, we added a new paragraph at the end that explicitly addresses the broader implications and future perspectives.** This addition emphasizes the need for integrative approaches combining morphometric and molecular analyses (e.g., SSRs, SNPs, NGS-based methods) to validate phenotypic diversity, unravel genetic structure, and guide breeding and conservation strategies under climate change scenarios. By doing so, we believe the *Conclusions* section now not only synthesizes the most significant findings but also provides a clear outlook for subsequent research directions and practical applications.

We trust that these revisions have improved the clarity and scientific impact of the *Conclusions* section in accordance with the reviewer’s valuable suggestion.

**Regards.**

**Response to Reviewer #3**

**Reviewer #3:**

Recommendation: Minor revision Morphological characterization evaluations, which form the basis of plant breeding studies, are always used as the first step in determining the richness level of genetic resources. In this research article carried out in Iran, some morphological characters were evaluated in materials obtained from a total of 108 wild accessions of Iris meda. The subject of the research article was found to be original. The morphological measurement results will be a resource for researchers working on Iris species. The number of samples are sufficient and statistical evaluations are sufficient. Some minor revisions are needed.

**Response to Reviewer #3**

We sincerely thank Reviewer #3 for the careful evaluation of our manuscript and for the constructive and encouraging comments provided. Your recognition of the originality of our research topic, the adequacy of the sample size, and the sufficiency of the statistical analyses is highly appreciated. We are grateful for your thoughtful feedback, which has helped us to further refine and strengthen the manuscript.

To facilitate the identification of the revisions, all changes made in response to your constructive comments and suggestions have been **highlighted in turquoise within the manuscript**.

**Reviewer #3:**

- Please provide the software used for generating Fig. 1.

It was provided. Please see Fig. 1.

- There is need to add hypothesis with clear cut objectives.

**Dear Reviewer #3,**

As Reviewer #2 also raised a similar comment and suggestion regarding this point, the corresponding revisions have already been incorporated into the **‘Introduction’** section. For clarity, these changes have **been highlighted in green** within the manuscript, and you may easily refer to the relevant part there.

Added & Revised.

**Reviewer #3:**

- Please add the lines to the correlation matrix if they are appropriate for you.

**Response to Reviewer #3 – Correlation Matrix**

**Dear Reviewer #3,**

We sincerely thank the reviewer for this thoughtful suggestion. We carefully considered the possibility of adding lines to the correlation matrix to delineate trait groupings. However, the primary aim of including the correlation matrix in our manuscript was to highlight the strength, direction, and statistical significance of the associations among the measured traits. As such, the use of numerical correlation coefficients (r values) together with significance levels (p-values) provides a clear and standardized representation of these relationships.

Adding lines within the matrix could create unnecessary visual complexity without providing additional statistical meaning, since the strength and direction of associations are already quantitatively defined. Furthermore, the trait interrelationships were extensively interpreted and supported through complementary multivariate analyses (PCA, regression, and clustering), which allowed us to visualize grouping patterns more effectively.

For these reasons, we have retained the standard tabular format for the correlation matrix without additional lines. We believe this approach preserves clarity, avoids redundancy, and ensures consistency with established practices in morphometric and plant genetic resource studies.

**Reviewer #3:**

- PCA analysis results should be explained according to positive and negative status.

**Response to Reviewer #3 – PCA Results**

**Dear Reviewer #3,**

We sincerely thank the reviewer for this valuable observation. We would like to clarify that in the revised manuscript, the PCA results have already been presented and interpreted in accordance with both positive and negative loadings of the traits. Specifically, the traits with high positive loadings were discussed as contributing factors to the separation of accessions along the principal component axes, whereas those with negative loadings were explained as contrasting descriptors driving variation in the opposite direction. This interpretation highlighted the dual contribution of traits—both positive and negative—in shaping the morphological divergence among the *I. meda* accessions.

Therefore, the PCA section not only reports eigenvalues and variance explained but also provides a detailed account of how positive and negative coefficients determined the positioning of genotypes in the biplot. We believe this presentation already aligns with the reviewer’s suggestion, as it clarifies the directionality of trait contributions and their role in the observed clustering patterns.

**Reviewer #3:**

- The conclusion should clearly convey the real need and necessity of this study.

**Response to Reviewer #3 – Conclusion**

**Dear Reviewer #3,**

As Reviewer #2 also raised a similar comment and suggestion regarding this point, the corresponding revisions have already been incorporated into the **‘Conclusion’** section. For clarity, these changes have been **highlighted in green** within the manuscript, and you may easily refer to the relevant part there.

Added & Revised.

**Regards.**
