# Supplementary material for: Multivariate analysis of Iris meda Stapf based on phenological and morphological characteristics
Source: PLoS One. 2025 Dec 1;20(12):e0336783. doi: 10.1371/journal.pone.0336783 (PMC12668513; doi:10.1371/journal.pone.0336783)
Supplement: Attachment — Submitted filename: Response to Reviewers -Second.docx [file pone.0336783.s006.docx]

**Response to the Editor**

**Dear Dr. José M. Alvarez-Suarez,**

- We would like to sincerely thank you for dedicating your valuable time to handling our manuscript and for your clear and constructive editorial guidance. We are particularly grateful for the fair, academic, and scientifically rigorous manner in which you have managed this process.

- Also, We sincerely thank Reviewers 1 & 3 for the time and effort dedicated for evaluating and accepting our manuscript.

**Regards.**

**Response to Reviewer #2**

We would like to express our deepest gratitude for the careful and thorough evaluation of our manuscript. The modifications have been highlighted in **green** within the manuscript.

**- Comment:** The probability values of Table 3 should be checked because there is a missing of astrick on the p-value. The authors should define the level of signifance (0.01, 0.05). Based on the level of signifance, the p-values arranged between 0.00-0.04 are considered as the significant status.

We sincerely thank the reviewer for this insightful observation.

**- Response:** It was provided. Please see below the Table 3.

**Regards.**
